# Mesenchymal stem cells for peripheral nerve injury and regeneration: a bibliometric and visualization study

**DOI:** 10.3389/fneur.2024.1420402

**Published:** 2024-08-05

**Authors:** Aikebaierjiang Aisaiti, Shalayiding Aierxiding, Kutiluke Shoukeer, Aikeremujiang Muheremu

**Affiliations:** ^1^Key Laboratory of Orthopedic Regenerative Medicine, Sixth Affiliated Hospital of Xinjiang Medical University, Ürümqi, Xinjiang, China; ^2^Beijing Darwin Cell Biotechnology Co., Ltd., Beijing, China

**Keywords:** mesenchymal stem cells, neural regeneration, Schwann cells, bibliometrics, peripheral nerve injury

## Abstract

**Objective:**

To use bibliometric methods to analyze the research hotspots and future development trends regarding the application of mesenchymal stem cells in peripheral nerve injury and regeneration.

**Methods:**

Articles published from January 1, 2013, to December 31, 2023, were meticulously screened using the MeSH terms: TS = (“Mesenchymal stem cells” AND “Peripheral nerve injury”) OR TS = (“Mesenchymal stem cells” AND “Peripheral nerve regeneration”) within the Web of Science database. The compiled data was then subjected to in-depth analysis with the aid of VOSviewer and Cite Space software, which facilitated the identification of the most productive countries, organizations, authors, and the predominant keywords prevalent within this research domain.

**Results:**

An extensive search of the Web of Science database yielded 350 relevant publications. These scholarly works were authored by 2,049 collaborative researchers representing 41 countries and affiliated with 585 diverse academic and research institutions. The findings from this research were disseminated across 167 various journals, and the publications collectively cited 21,064 references from 3,339 distinct journals.

**Conclusion:**

Over the past decade, there has been a consistent upward trajectory in the number of publications and citations pertaining to the use of mesenchymal stem cells in the realm of peripheral nerve injury and regeneration. The domain of stem cell therapy for nerve injury has emerged as a prime focus of research, with mesenchymal stem cell therapy taking center stage due to its considerable promise in the treatment of nerve injuries. This therapeutic approach holds the potential to significantly enhance treatment options and rehabilitation prospects for patients suffering from such injuries.

## 1 Introduction

Peripheral Nerve Injury (PNI) is a prevalent neurological condition encountered in clinical practice, frequently induced by a spectrum of physical traumas such as road traffic accidents, construction mishaps, natural disasters, combat injuries, athletic incidents, injectable drug-related trauma, and electrical injuries (1, 2). The global annual incidence of PNI is approximated to fall within the range of 13 to 23 cases per 100,000 individuals (3–5). PNI is notorious for its challenging treatment landscape, unfavorable prognoses, high incidence of disability, and the considerable pain and psychological distress it inflicts on patients and their families (6, 7). While conventional treatment modalities encompass surgical intervention, physical therapy, and pharmacological treatments, these approaches often fall short of fully restoring functionality in many instances (8, 9). Consequently, the pursuit of novel therapeutic strategies to enhance the repair and regeneration of peripheral nerves assumes paramount significance (10, 11). Emerging from the horizon of regenerative medicine, the utilization of mesenchymal stem cells (MSCs) has demonstrated potential in differentiating into neural cells to treat PNI, thereby presenting novel avenues for augmenting patient treatment and rehabilitation (12, 13). This innovative approach has ignited optimism within the academic and medical communities, as well as among patients, regarding the potential for improved treatment outcomes and enhanced quality of life (14, 15).

MSCs are ubiquitously distributed within a plethora of tissues, including bone marrow, umbilical cord blood, the subendothelial layer of umbilical veins, adipose tissue, peripheral blood, and muscle (16, 17). As stem cells, MSCs possess a constellation of advantages, including their widespread availability, ease of accessibility, low immunogenicity, potential for osteogenic differentiation, and their capacity for robust proliferation and self-renewal (18, 19). Consequently, they are esteemed as the quintessential “seed cells” within the domain of tissue engineering (20, 21). In recent times, MSCs have garnered significant attention and support from researchers across various disciplines, including bone tissue engineering, peripheral nerve injury repair, and regeneration, with a particular emphasis on the latter (22–25). This therapeutic approach holds immense promise due to MSCs’ potential to differentiate into nerve cells, thereby bolstering neural regeneration, enhancing the prognosis for patients with peripheral nerve injuries, reducing disability rates, alleviating pain, and diminishing the burden on patients and their families (26–28). However, the effectiveness and safety of this treatment method require further validation through rigorous research and clinical trials. In this study, a bibliometric approach was adopted, leveraging the Web of Science database to compile and select pertinent literature on MSCs within the context of peripheral nerve injury and regeneration, spanning from January 2013 to December 2023. Through a multifaceted visualization analysis of the aggregated literature, encompassing an examination of contributing countries, authors, institutions, journals, articles, and keywords (29), the objective is to offer insights into the prevailing research foci and emerging development trends in this domain. The purpose of current study was to chart new pathways for the treatment of PNI with the aim of maximizing benefits, and to furnish the scientific community with empirical data, and theoretical underpinnings to inform future research endeavors.”

## 2 Materials and methods

### 2.1 Data collection and retrieval

A total of 360 articles related to MSCs and PNI were extracted from the Web of Science database, which provided a core collection of relevant literature. These publications were thoroughly reviewed according to unified standards, resulting in the confirmation of 350 valid articles and reviews. These publications were utilized for co-authorship, co-journal, co-institution, and co-country network analysis, along with citation analysis. To mitigate errors arising from database updates and to minimize the subjectivity of different screeners’ selections, a single researcher completed the screening and collection of all the articles on December 31st, 2023. This approach established a robust data foundation for subsequent research analysis, thereby elucidating the cooperative networks and cutting-edge research trends within the domains of MSCs and PNI.

#### 2.1.1 Retrieval method and search terms

In the advanced search function of the Web of Science database, we delineated our search within the Core Collection, targeting the specific topics of “Mesenchymal stem cells” and “Peripheral nerve injury” or “Mesenchymal stem cells” and “Peripheral nerve regeneration.” The search query was constructed as Topic = (“Mesenchymal stem cells” AND “Peripheral nerve injury”) OR Topic = (“Mesenchymal stem cells” AND “Peripheral nerve regeneration”). Following a meticulous screening of the retrieved literature, we compiled a comprehensive plain text file that encapsulated the complete bibliographic records and references. Subsequently, this data was processed through the visualization software Cite Space 6.4.2R for a detailed analysis. Furthermore, supplementary research analysis was conducted utilizing both Cite Space and VOSviewer. The literature search began on January 1, 2013, and ended on December 31, 2023, covering the “article” and “review” categories. This section offers a comprehensive narrative of the search methodology and the specific search terms employed, thereby aiding other researchers in comprehending the research approach and facilitating further studies within related disciplines.

**Table:** 

#1 subject term: Mesenchymal stem cells
#2 free word: Peripheral nerve injury OR Peripheral nerve disease;
#3 #2 AND #1
#4 subject term: Peripheral nerve regeneration;
#5 subject term: treatment;
#6 #3 AND #4 AND #5

#### 2.1.2 Inclusion and exclusion criteria

The inclusion criteria for this study were confined to article and review pertinent to the application of MSCs in the treatment and regeneration of peripheral nerves, sourced from the Web of Science database between January 1st, 2013, and December 31st, 2023. The exclusion criteria were meticulously defined to include: (1) duplicate literature, (2) non-original content such as notifications, comments, translations, conference papers, abstracts, newspaper articles, patents, news reports, lectures, autobiographies, and graduate theses. The establishment of these stringent criteria was instrumental in ensuring the consistency and quality of the literature selection process, thereby enhancing the reliability of the resulting corpus.

#### 2.1.3 Data analysis

The Web of Science database was used to analyze data, extracting key information such as publication year, country/region, author, and other relevant factors from the selected literature. Visualization analysis tools such as Cite Space, VOSviewer, and Microsoft Excel 2019 were then employed for data visualization. Cite Space is a powerful bibliometric tool capable of analyzing citation networks, keyword networks, and author collaboration networks, among others, providing a deeper understanding of frontiers, hotspots, and trends in the research field. On the other hand, VOSviewer specializes in visualizing bibliometric networks, grouping related nodes and using different colors to distinguish clusters, thereby helping researchers discover patterns and relationships in large-scale bibliographic data. These two software programs have been widely used in bibliometric research to aid in better understanding bibliographic data visually. In this study, we used them to analyze various aspects of literature, such as annual distribution, country/region distribution, author cooperation networks, and keyword networks, to generate knowledge maps, keyword clustering maps, and other visual charts in order to comprehensively study and analyze the development trends and key nodes in the research field. This will help reveal cutting-edge research directions and provide valuable insights for researchers.

## 3 Results

**Figure 1:**
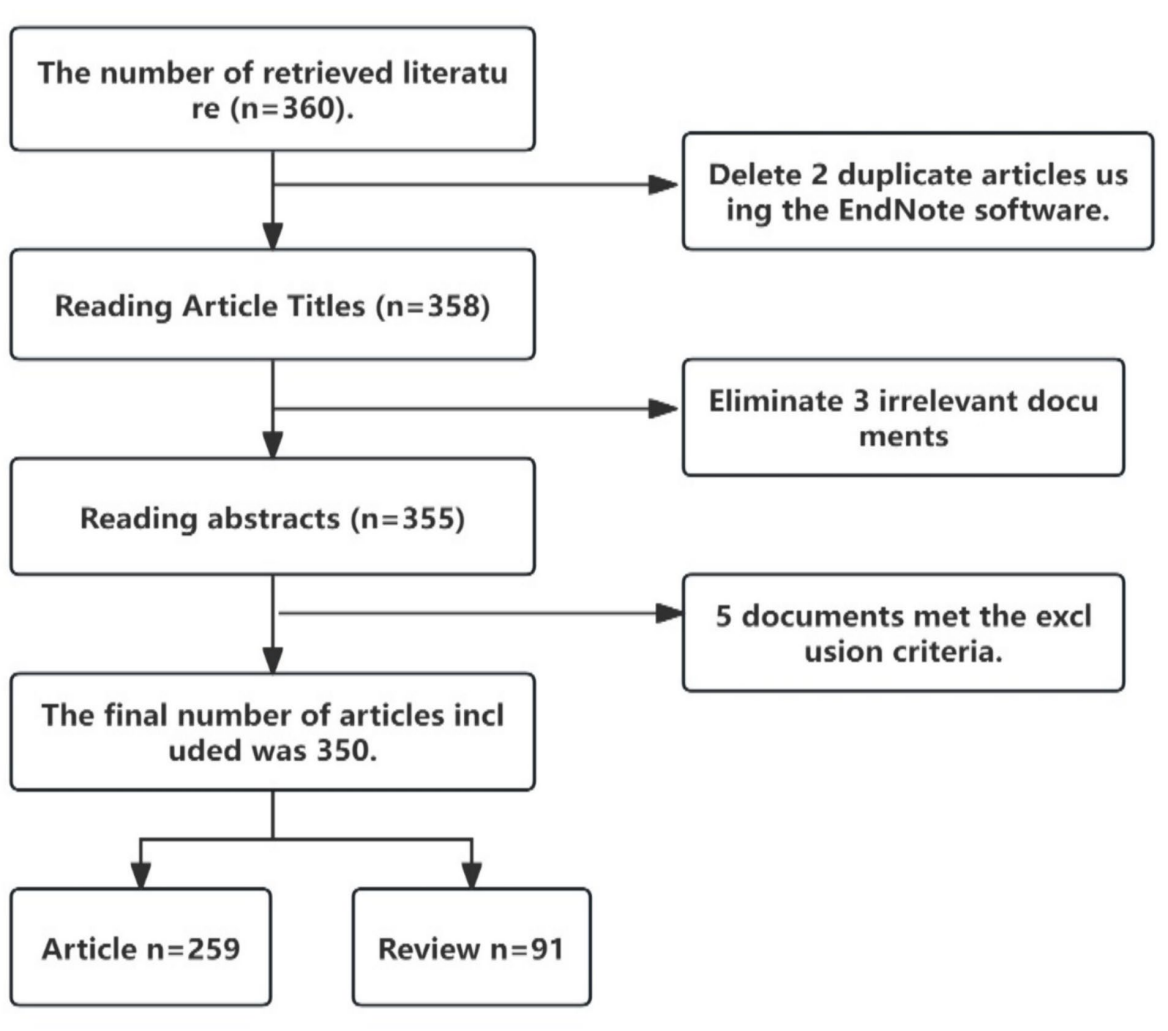
Flowchart of literature search and screening process.

We collected a total of 360 pieces of literature from the Web of Science database. After conducting a thorough review and eliminating some of the gathered literature, we ultimately chose 350 publications for inclusion in this study, as shown in Figure 1. These 350 publications were authored by 2049 individuals from 41 different countries and 585 institutions. They were published in 167 distinct journals and collectively referenced a total of 21,064 publications from 3,339 journals.

### 3.1 Global trend in article publications

**Figure 2:**
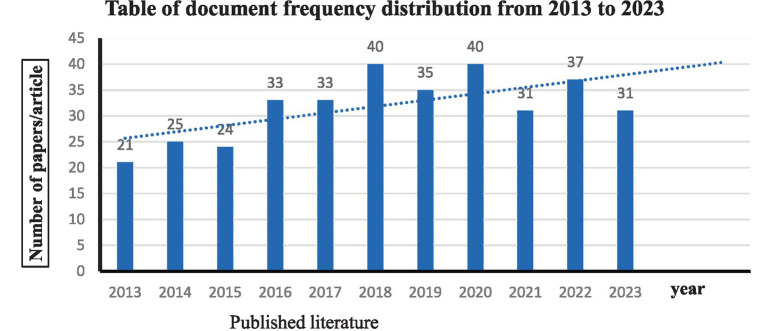
The annual distribution of publications in the Web of Science database.

The volume of literature publications serves as a critical metric for gauging the evolving trends and emerging focal points within a research domain. By examining the global literature output over the past decade in the field of MSC therapy for peripheral nerve injury and regeneration within the Web of Science database, we can discern shifts in research orientation and the developmental trajectory of this area. This analysis offers crucial insights into the research hotspots, thereby enriching our understanding of the field. Figure 2 encapsulates the publication status of literature in this domain over the past decade, detailing the retrieval of a total of 350 publications and an average annual output of approximately 32 literature. The Figure 2 reveal a consistent trend where the annual literature publication count has consistently exceeded 20 publications from 2013 to 2023. Notably, there were six years with a publication volume surpassing the average (31.8 literature), with 2018 and 2020 standing out as the pinnacles with 40 articles each. Despite annual fluctuations in publication counts, the overall pattern suggests a robust level of annual publications, with a minimum of 20 articles published annually in this field. It is noteworthy that, although the literature publication count for 2023 stands at 31, given the historical trend, it is highly probable that the total will surpass 31 by the year’s end (Figure 2).

### 3.2 National distribution

**Figure 3:**
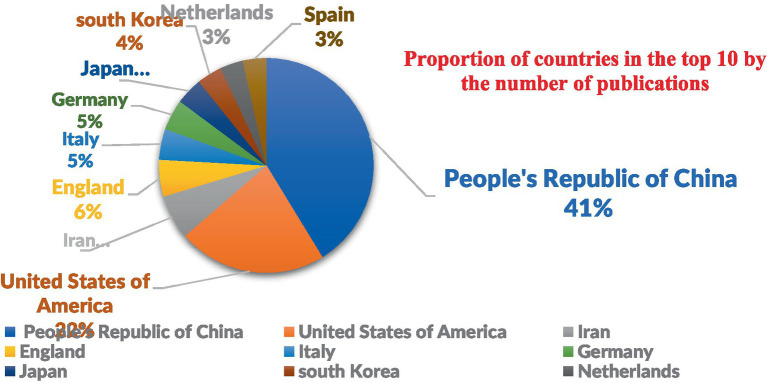
Proportion of the top 10 countries in the field of academic literature publications.

**Figure 4:**
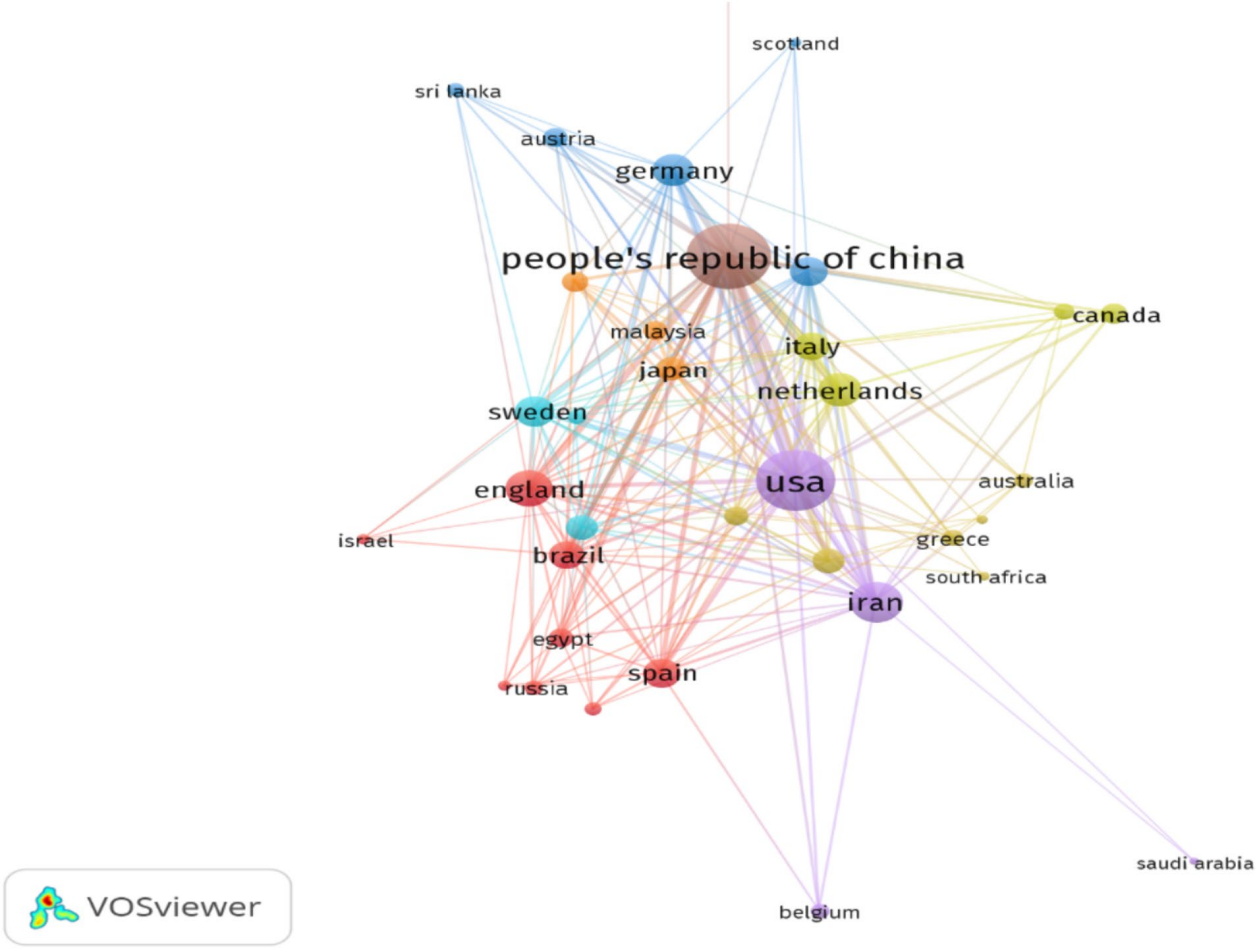
National collaborative analysis of studies related to using mesenchymal stem cells for the treatment of peripheral nerve injury and regeneration.

**Figure 5:**
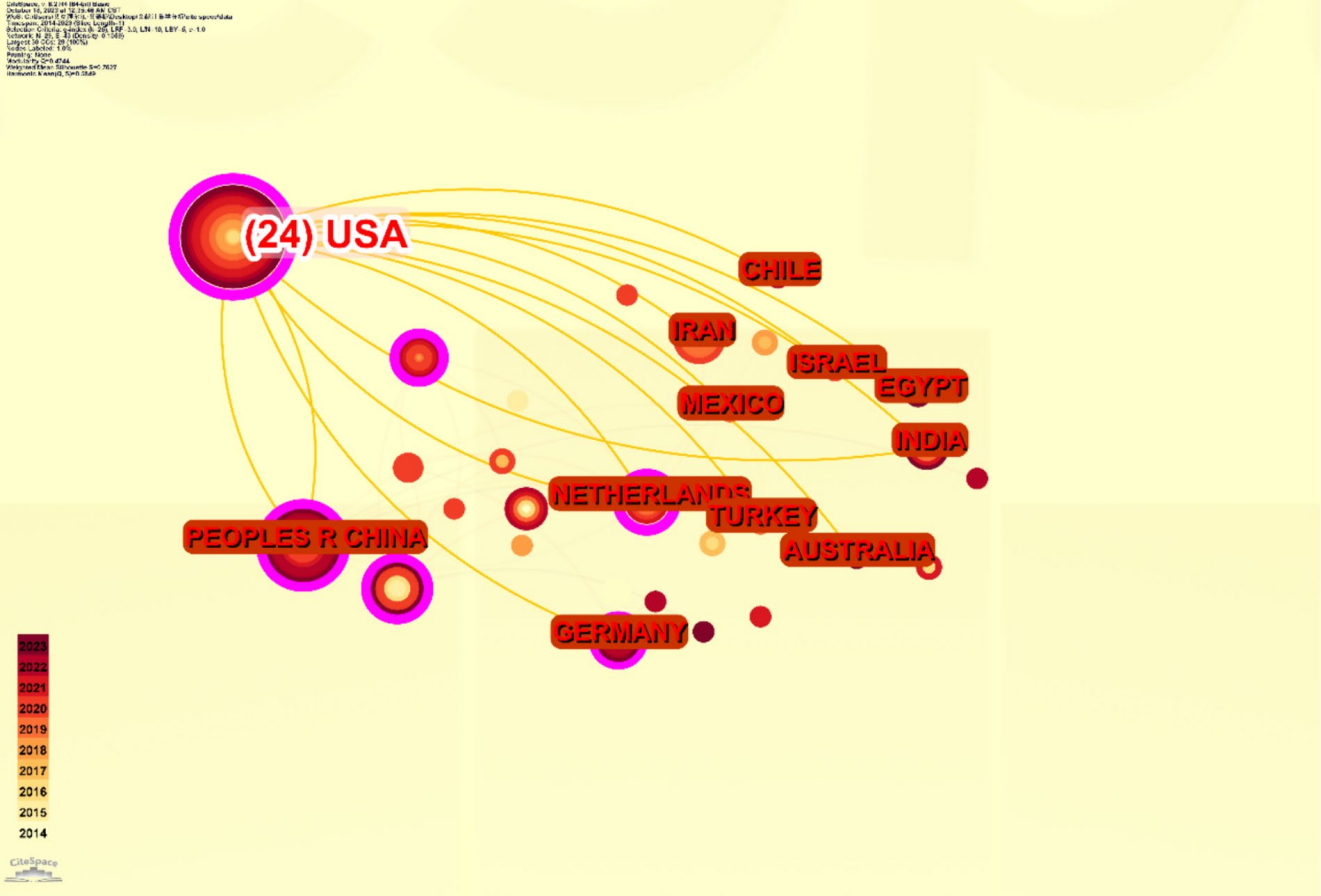
Chart of cooperative relations among various countries.

**Figure 6:**
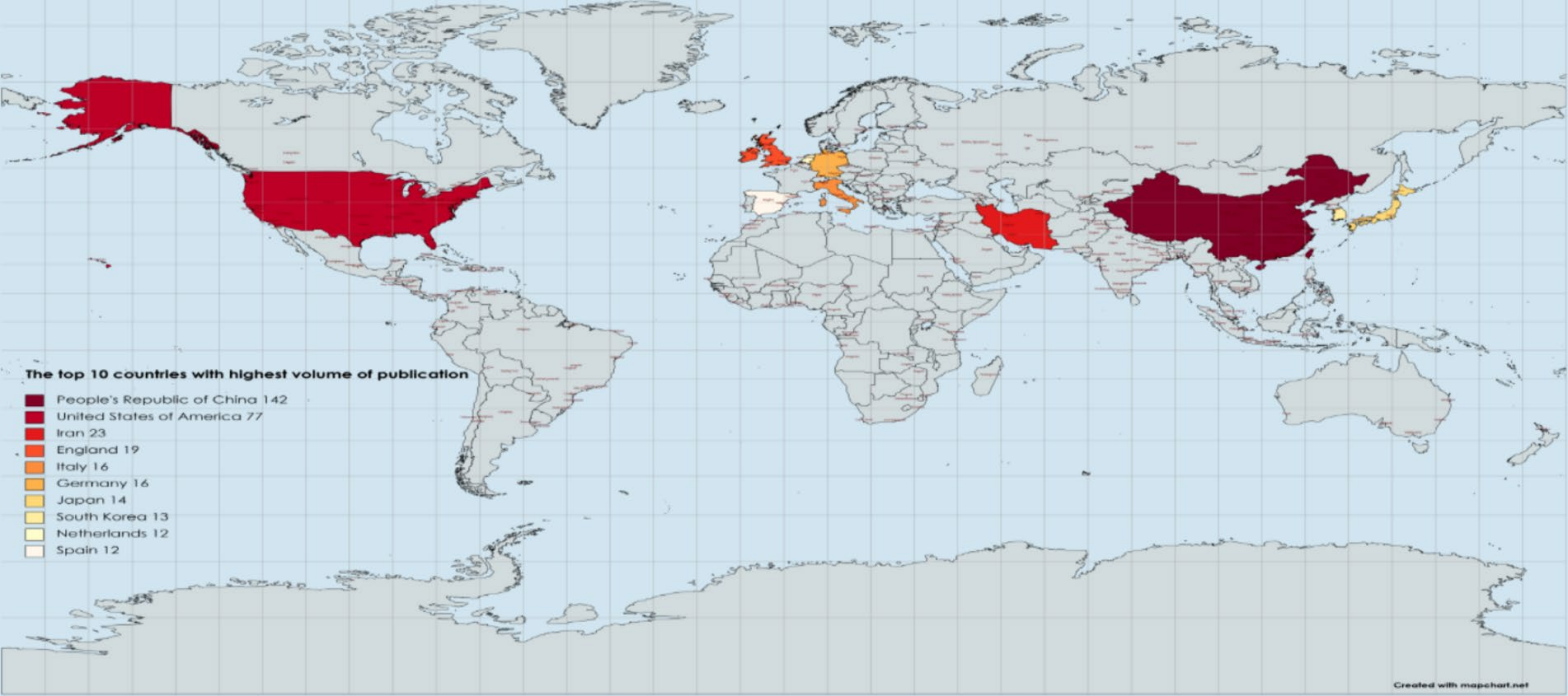
Global distribution of the top 10 countries in terms of published literature.

Among the 40 distinct countries and regions represented, Figure 3 illustrates that China leads in terms of publication output, with a total of 142 papers, comprising 41.0% of the global total. The United States follows closely with 77 papers, accounting for 22.0% of the total. Collectively, these two nations account for 63.0% of the global publication output, underscoring their substantial influence in the field of mesenchymal stem cell therapy for peripheral nerve injury and regeneration (Figure 3). In the VOSviewer visualization (Figure 4), the size of each node corresponds to its frequency of occurrence, and the density between nodes indicates the level of association (Figure 5). Figure 6 demonstrates the geographical distribution of the top ten countries in terms of global publication output, suggesting a network of collaborative relationships among China, the United States, the United Kingdom, Germany, and other nations. This suggests that research in this domain has become a global academic endeavor. Cite Space visualization analysis reveals that the United States maintains the closest connections with other countries and holds a significant academic influence in this field (Figure 5).

### 3.3 Author distribution

Among the 350 publications within this domain, a total of 2049 authors contributed to their publication. From 2013 to 2023, authors across the globe collaborated to produce 350 English-language literature on MSCs therapy for peripheral nerve injury and nerve regeneration. Table 1 details the top 10 authors, who collectively authored 62 articles, representing approximately 17.71% of the total. These authors hail from diverse nations including China, the United States, Sweden, Japan, and Portugal, and have collectively advanced the field with their substantial contributions. Dr. PENG J from Nantong University stands out with 10 publications, contributing 2.86% of the total and ranking first. Dr. WANG Y follows with 7 articles, representing 2.0% of the total and ranking second, underscoring the significant academic influence of Nantong University in this domain (Table 1).

**Table 1:** Co-authorship analysis of the top 10 authors in terms of number of publications.

Author	Publication (NO)	Proportion (%)	Citations (times)	Country	Institution
Peng J	10	2.86	221	China	Nantong University
Wang Y	7	2.0	156	China	Nantong University
Sakaguchi	6	1.71	196	USA	Iowa State University
Gu XS	6	1.71	196	China	Nantong University
Yang YM	6	1.71	275	China	Nantong University
Kingham PJ	6	1.71	587	Sweden	University of Umea
Shin A	6	1.71	88	USA	Mayo School of Medicine
Malla PS	5	1.43	127	USA	Iowa State University
Ikeguchi R	5	1.43	139	Japan	Kyoto University
Mauricio AC	5	1.43	108	Portugal	University of Porto

### 3.4 Distribution of institutions

**Figure 7:**
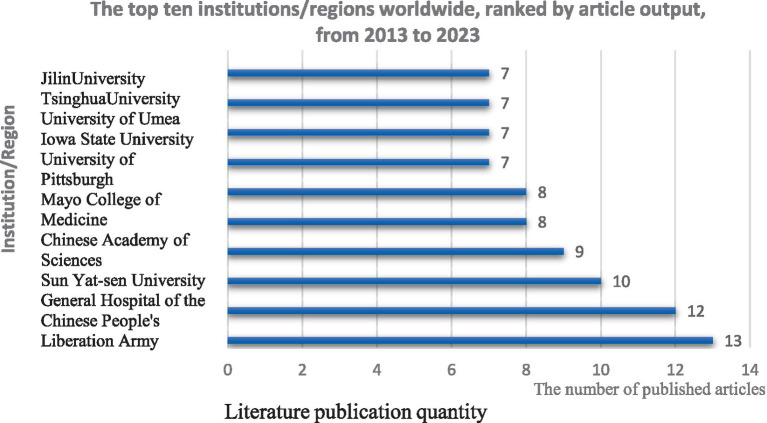
Top 10 institutions and regions ranked by the number of publications in the field.

**Figure 8:**
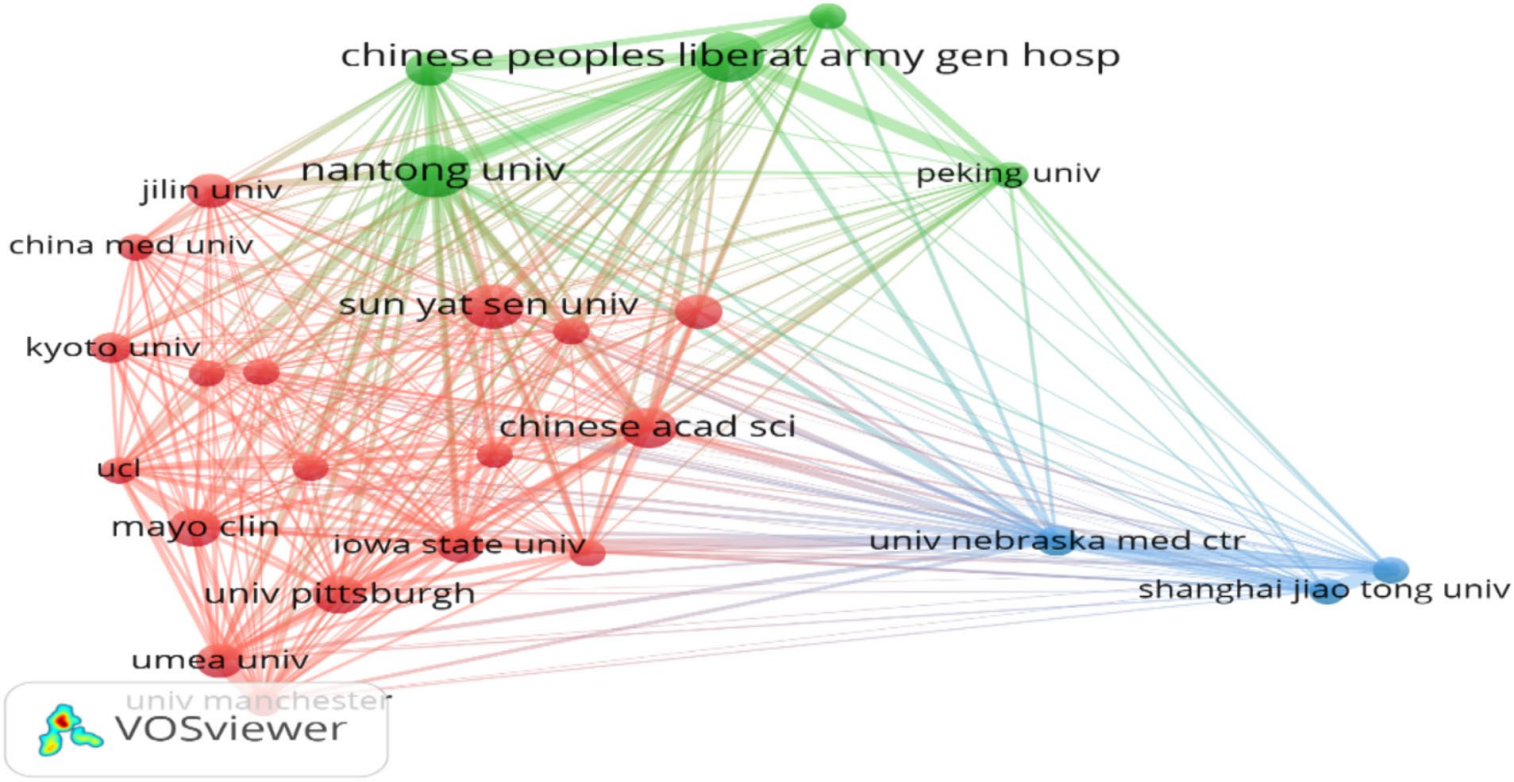
Co-authorship analysis of literature institutions.

In the domain of MSCs therapy for peripheral nerve injury and regeneration, a total of 585 institutions and organizations worldwide are actively engaged, indicating a vibrant landscape of international collaboration and activity. Among these, 26 institutions/regions have published more than five articles. Figure 7 delineates the top 10 institutions/organizations in terms of publication volume. Nantong University in China leads the pack with 13 publications, closely followed by the Chinese PLA General Hospital and Sun Yat-sen University, each with 12 and 10 publications, respectively. This underscores the robust research activity in this field in China (Figure 7). Clustering analysis of these institutions conducted using VOSviewer software illuminates the collaborative relationships and research focal points among institutions/organizations (Figure 8). The size of the nodes in the figure corresponds to the number of articles published by each institution, with larger nodes indicating a greater number of publications. Additionally, the thickness of the lines connecting the nodes denotes the frequency of collaboration between the represented institutions. Consequently, from Figure 8, it is evident that there is a robust network of connectivity among various institutions/organizations within China, as well as between China and other global research institutions. This collaborative network fosters knowledge exchange and cooperation, contributing to the advancement of research on peripheral nerve injury and regeneration (Figure 8).

### 3.5 Journal analysis

**Figure 9:**
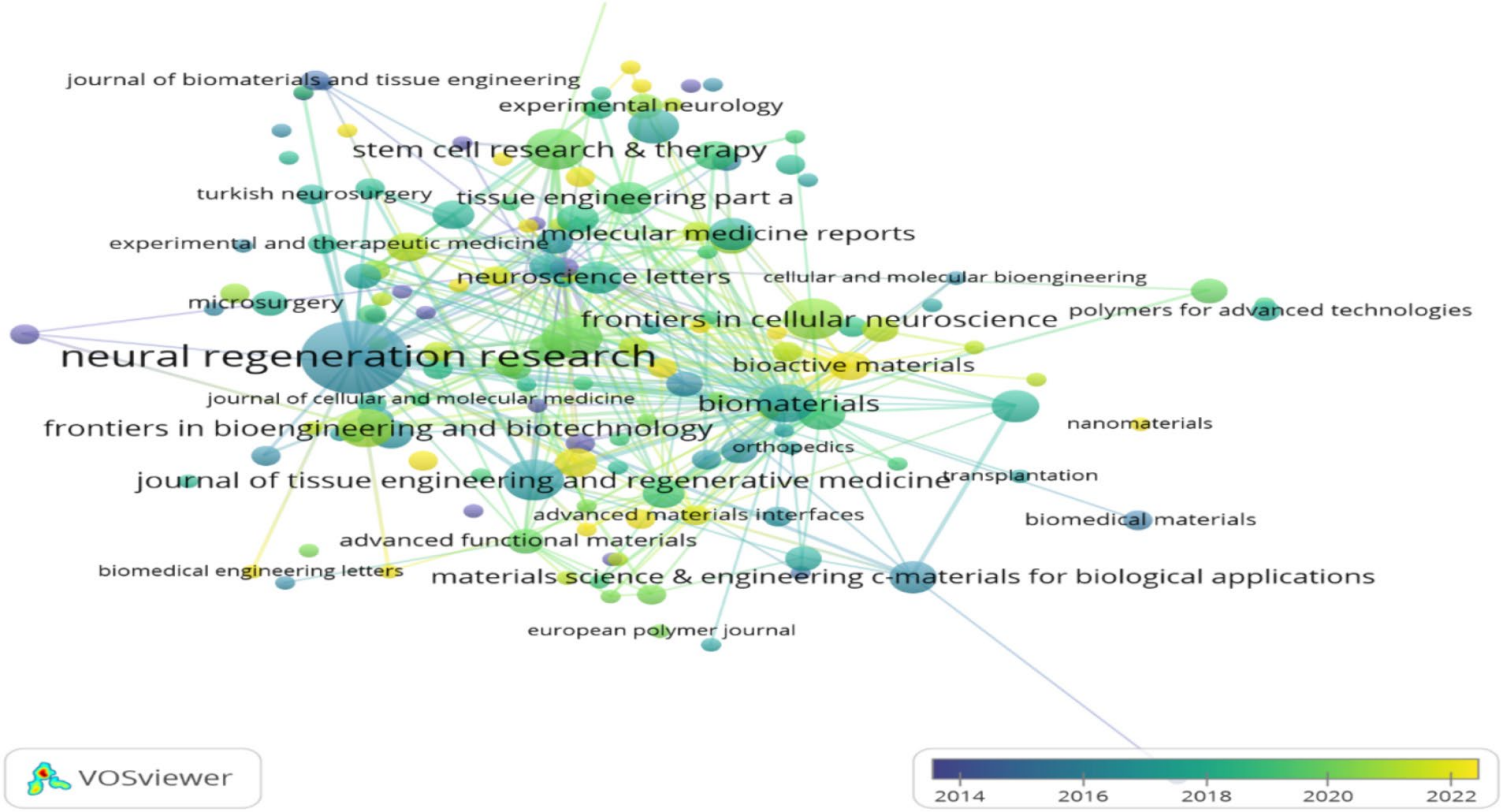
An analysis of publication time in the journal.

Over the past decade, the top ten journals in the literature on mesenchymal stem cell therapy for peripheral nerve injury and regeneration research are listed in Table 2. The top three journals are Neural Regeneration Research, International Journal of Molecular Sciences, and Stem Cell Research Therapy, with 25, 8, and 8 publications, respectively. Notably, the impact factor of the Biomaterials journal reached 15.3 in 2022–2023, with an average citation of 96.57 times per article. Among the top ten journals, it consistently ranks first in both impact factor and average citation, signifying its preeminence in the field (Table 2). Figure 9 presents a visual analysis of the publication journals in this field from 2013 to 2023 using the publishing journal analysis feature of VOS viewer software. In the figure, a color-coded scale line is arranged from dark to light in the lower right corner. The closer the circle color is to the dark, the more likely authors are to submit and publish their work in earlier academic journals. Conversely, the closer it is to light, the more likely it is to represent current research hotspots (Figure 9).

**Table 2:** The ranking of the top 10 journals in terms of the global publication output in the field.

Rank	Journal	Publication (NO)	Citations (times)	Average citations (times)	IF (2022)
1	Neural Regeneration Research	25	504	20.16	6.0
2	International Journal of Molecular Sciences	8	172	21.5	6.2
3	Stem Cell Research Therapy	8	362	45.25	8.0
4	Journal of Tissue Engineering and Regenerative Medicine	8	312	39.0	4.3
5	Frontiers in Cellular Neuroscience	8	138	17.25	6.1
6	Biomaterials	7	676	96.57	15.3
7	Frontiers in Bioengineering and Biotechnology	7	325	46.42	5.7
8	Plos One	6	127	21.2	3.7
9	Acta Biomaterialia	5	205	41.0	9.7
10	Tissue Engineering Part A	5	84	16.8	4.0

### 3.6 Analysis of cited references and citations

**Figure 10:**
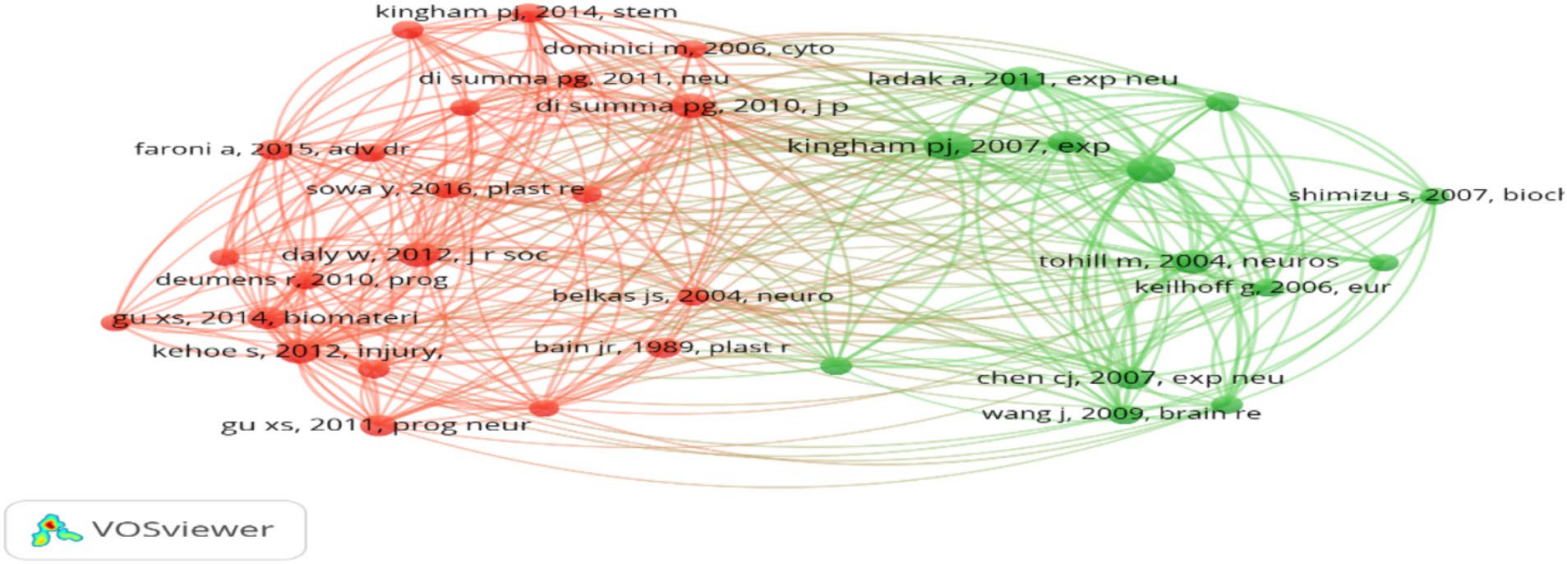
Analysis of cited reference.

Among the 21,064 cited references included in the study, a screening criterion was established for documents that had been cited at least 20 times. As a result, 34 references were selected for inclusion, as depicted in Figure 10. In the figure, each circle symbolizes a distinct cluster, with the number of circles indicating the number of references analyzed. The size of each circle represents the number of citations each reference has received. Two interconnected dots indicate that two references were cited by the same paper. The length of the connecting lines between the dots indicates the level of correlation between the cited references – the shorter the line, the stronger the correlation (Figure 10).

### 3.7 Literature analysis

**Figure 11:**
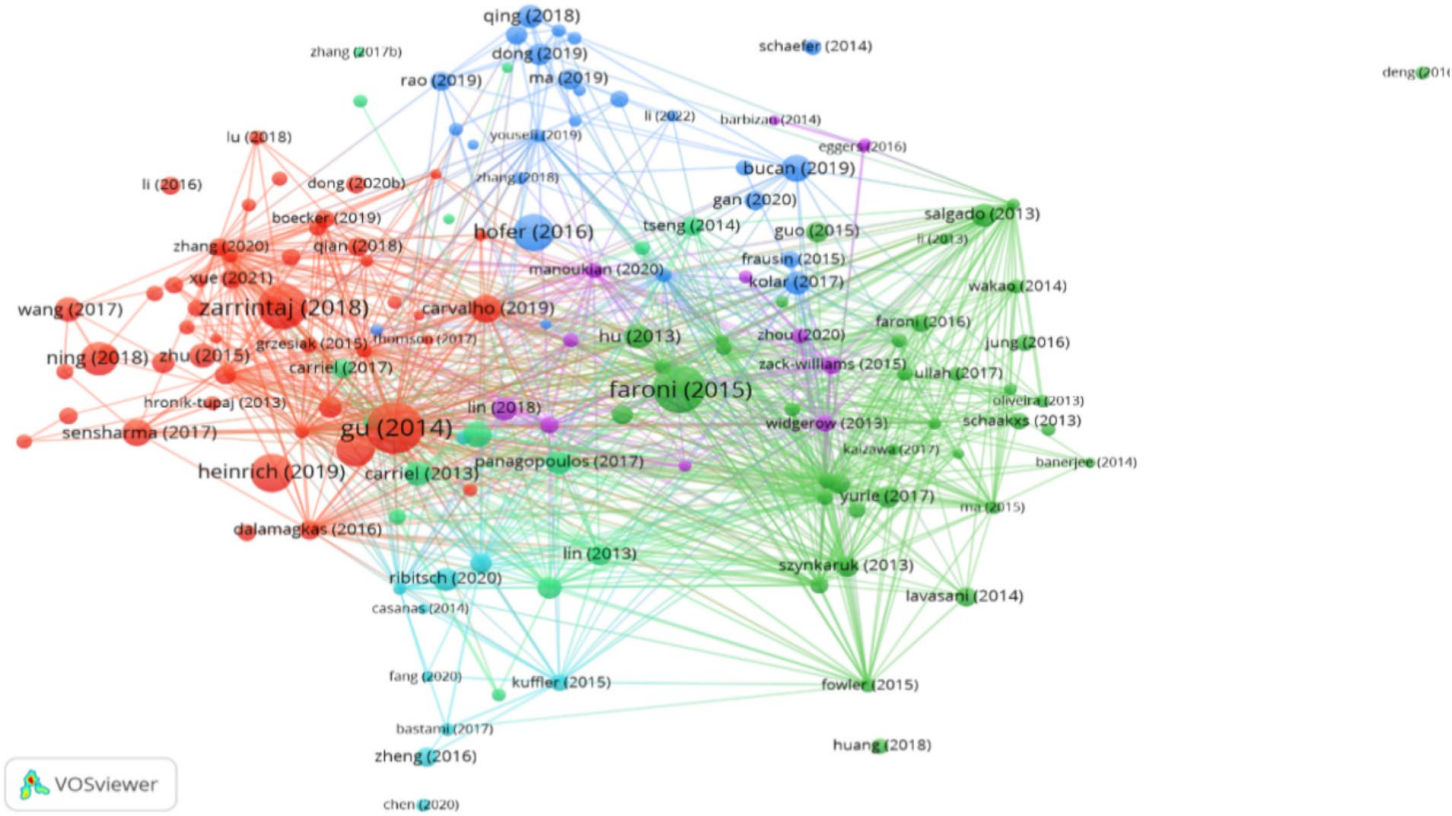
Distribution analysis of literature citations.

Utilizing the VOSviewer software for citation analysis, we identified the most highly cited papers (Figure 11) and the top 10 most cited papers (Table 3) in the domain of mesenchymal stem cell therapy for peripheral nerve injury and regeneration research from 2013 to 2023. In Figure 11, the distinct colors of the circles represent different clusters, and the size of the circles denotes the number of times each paper has been cited. Larger circles signify a higher level of academic influence within the field (Figure 11). Table 3 reveals that the most cited paper is “Neural Tissue Engineering Options for Peripheral Nerve Regeneration” by the Chinese author “GU XS,” published in the journal Biomaterials in 2014, and has been cited 422 times. The second most cited paper is “Peripheral Nerve Regeneration: Experimental Strategies and Future Perspectives” with “Faroni” as the first author, published in the journal Advanced Drug Delivery Reviews in 2015, and has been cited 372 times. The third most cited paper is “Agarose-based Biomaterials for Tissue Engineering” by “Zarrintaj” as the first author, published in the journal Carbohydrate Polymers in 2018, and has been cited 333 times (Table 3). The keywords of these highly cited papers are concentrated in the fields of “mesenchymal stem cells,” “peripheral nerve injury,” and “peripheral nerve regeneration.”

**Table 3:** Top 10 cited articles on mesenchymal stem cell therapy for peripheral nerve injury and regeneration research (2013–2023).

Rank	Title	Citations (times)	First author	Journal
1	Neural Tissue Engineering Options for Peripheral Nerve Regeneration	422	GU XS	Biomaterials
2	Peripheral Nerve Regeneration: Experimental Strategies and Future Perspectives	372	Faroni	Advanced Drug Delivery Reviews
3	Agarose-based Biomaterials for Tissue Engineering	333	Zarrintaj	Carbohydrate Polymers
4	3D Bioprinting: from Benches to Translational Applications	234	Heinrich	Small
5	Secreted Trophic Factors of Mesenchymal Stem Cells Support Neurovascular and Musculoskeletal Therapies	229	Hofer	Stem Cell Research Therapy
6	Collagen – Emerging Collagen-Based Therapies Hit the Patient	195	Abou NL	Advanced Drug Delivery Reviews
7	Electroactive Polymers for Tissue Regeneration: Developments and Perspectives	179	Ning	Progress in Polymer Science
8	Modern Trends for Peripheral Nerve Repair and Regeneration: Beyond the Hollow Nerve Guidance Consult	135	Carvalho	Frontiers in Bioengineering and Biotechnology
9	Biomaterials and Cells for Neural Tissue Engineering: Current Choices	124	Sensharma	Materials Science Engineering c-Materials for Biological Applications
10	Effect of Exosomes Trom Rat Adipose-Derived Mesenchyma Stem Celis on Neurite Outgrowth and Sciatic Nerve Regeneration Antercrush Injury	119	Bucan	Molecular Neurobiology

### 3.8 Keyword analysis

**Figure 12:**
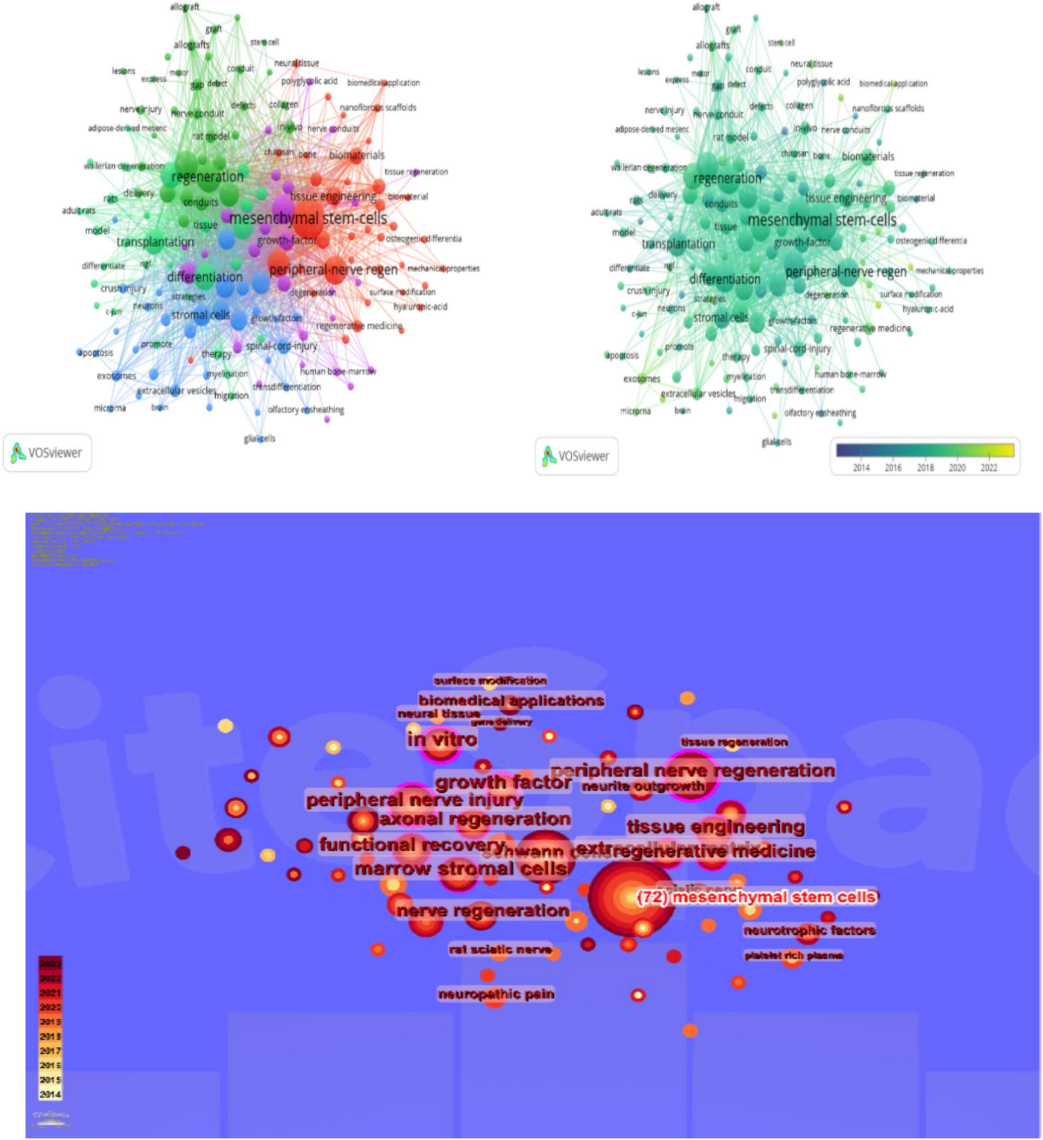
**(A)** Distribution analysis of keywords. **(B)** Time distribution analysis chart of keywords in the field. **(C)** Distribution of keywords in the field of mesenchymal stem cells for peripheral nerve injury and regeneration.

**Figure 13:**
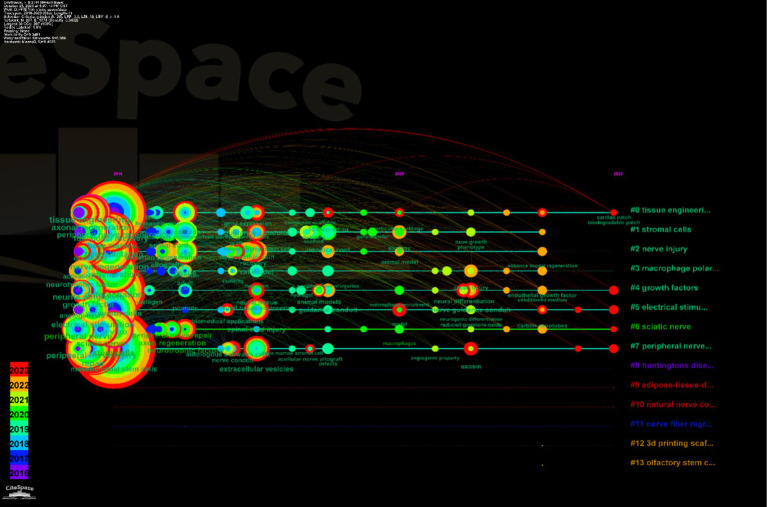
Distribution chart of the temporal analysis of keywords in this field.

By leveraging the VOSviewer software for keyword analysis, we compiled a total of 1,689 keywords from the literature on mesenchymal stem cell therapy for peripheral nerve regeneration research. Among these, 162 keywords appeared five times or more. “Mesenchymal stem cells” was the most frequently occurring keyword, appearing a total of 163 times, with an association strength of 1,197. The second-ranked keyword was “regeneration,” which appeared 87 times across all keywords, with an association strength of 696. The top five keywords also included “Schwann-Cells” (86 occurrences), “Repair” (85 occurrences), and “Peripheral-Nerve Regeneration” (84 occurrences). This underscores the centrality of these five keywords “Mesenchymal stem cells,” “Regeneration,” “Schwann-cells,” “Repair,” and “Peripheral-Nerve Regeneration”—in the conceptual landscape of this field (Table 4). Through VOSviewer analysis (Figure 12A), we categorized these 162 keywords with a frequency of 5 or more into four clusters. In the figure, each circle represents a keyword, and the size of the circle reflects the frequency of occurrence of that keyword. The larger the circle, the higher the frequency of occurrence of that keyword in the research field, signifying its greater importance within that domain of study (Figure 12A). The colors in Figures 12B,C reflect the frequency of the keywords. The keyword’s appearance in Figure 12B is indicated by the darkness of the color, with darker colors representing earlier appearances and lighter colors indicating recent popularity. From these figures, it is evident that “Mesenchymal stem cells,” “Regeneration,” “Schwann-cells,” “Repair,” and other keywords have garnered significant attention in recent research and have emerged as research focal points (Figures 12B,C). Finally, we employed the timeline of these keywords to track their evolving popularity over time (Figure 13), which vividly illustrates the temporal relationship between keywords (Figure 13).

**Table 4:** Top ten keywords ranked by frequency of occurrence.

Rank	Keywords	Frequency (times)	Link strength (times)
1	Mesenchymal Stem-Cells	163	1,197
2	Regeneration	87	696
3	Schwann-cells	86	766
4	Repair	85	682
5	Peripheral-Nerve Regeneration	84	569
6	*In-vitro*	76	607
7	Differentiation	75	624
8	Peripheral Nerve Injury	70	580
9	Nerve Regeneration	64	542
10	Transplantation	63	533

### 3.9 Journal outbreak analysis

**Figure 14:**
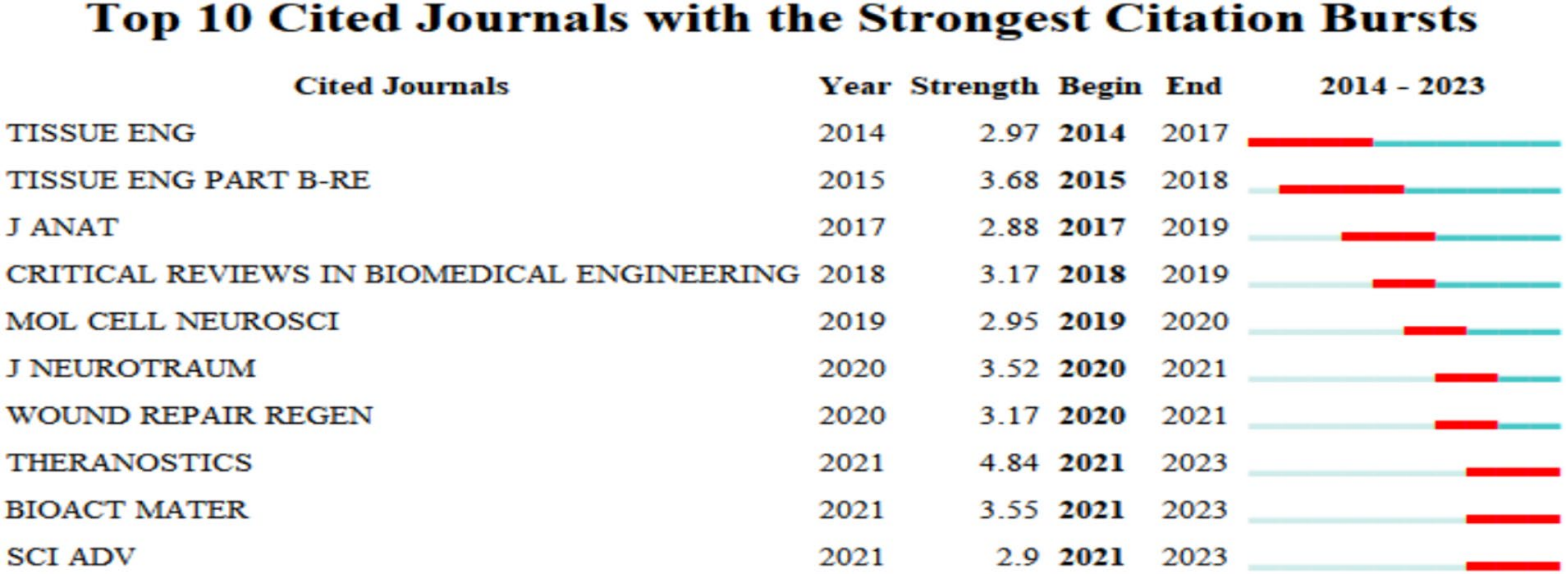
Journal outbreak analysis of the main publications in this field.

In Figure 14, we depict the top ten journals ranked based on a timeframe from 2013 to 2023, with a minimum outbreak intensity standard of 2.9. This ranking reflects a prevailing trend, with the journal “THERANOSTICS” emerging in 2021 and ascending in prominence until 2023, achieving an outbreak intensity of 4.84 and securing the top position. This ascent was sustained for two consecutive years. Trailing in second place is “TISSUE ENG PART B-RE,” which emerged between 2015 and 2018, maintaining a continuous outbreak for a minimum of 3 years and registering an outbreak intensity of 3.68. This data suggests that these 10 journals are frequently chosen by researchers and are highly esteemed by the academic community (Figure 14).

## 4 Discussion

In this study, we employed bibliometric analysis to systematically examine the research trends in mesenchymal stem cell therapy for peripheral nerve injury and regeneration from 2013 to 2023. We delineated the developmental patterns over this period, encompassing the annual publication count, geographical distribution, the top 10 authors by publication volume, the top 10 cited articles, and the top 10 institutions by publication volume. Furthermore, we conducted a polynomial integration analysis on the cumulative publication volume per year. The Web of Science database, renowned for its high quality and reliability, served as our primary data source. Initially, a total of 360 publications were retrieved through extensive searches within the Web of Science database. Following meticulous screening and elimination, 350 publications were identified as pertinent to the research subject. These publications represent a broad spectrum of research disciplines, all dedicated to advancing the understanding and application of mesenchymal stem cell therapy for peripheral nerve injuries and regeneration.

Our study’s findings revealed that China currently holds a preeminent position in both the quantity and quality of publications and practical applications within the domain of mesenchymal stem cell therapy for peripheral nerve injury and regeneration. This may be attributed to several factors, including its vast population and the substantial number of patients with peripheral nerve injuries annually due to diverse reasons. Additionally, China’s robust research and development ecosystem provides a conducive platform for such studies. According to the analysis of the funding support from all over the world through the application of VOSviewer, the National Natural Science Foundation of China has significantly augmented its investment in research within this field, which has collectively bolstered China’s research level and capabilities in mesenchymal stem cell therapy for peripheral nerve injury and regeneration.

While there is close internal cooperation among Chinese scholars and institutions, United States is still the leading country in terms of international collaboration, with a relatively scattered network of collaboration. Studies have shown that strengthening international collaboration between authors, institutions, and countries helps to improve the impact of scientific research papers. Therefore, countries should continue to actively engage in international collaboration, and relevant institutions should provide more channels for collaboration, encourage researchers to actively participate in international research cooperation to share knowledge, leverage their respective strengths, and address shortcomings to make greater progress in this field to promote the research advancement in the field of mesenchymal stem cell-based treatment of peripheral nerve injury.

In this analysis, we employed two academic analysis tools, Cite Space and VOS viewer, to systematically review the research development trends of mesenchymal stem cell therapy for peripheral nerve injury and regeneration from January 2013 to December 2023 within the Web of Science database. Over the past decade, the global academic publication volume in this field has exhibited a relatively stable trend, with an annual output consistently exceeding 20 articles. This sustained output reflects the growing interest from scholars in addressing the challenges of peripheral nerve injury and regeneration repair.

Peripheral nerve injury (PNI) is a prevalent clinical neurological condition, characterized by a high incidence rate and posing significant management challenges (30–32). The prognosis for PNI is typically poor, leading to a high incidence of disability. Patients and their families often endure prolonged physical and psychological suffering, accompanied by a considerable economic burden (33, 34). Worryingly, the incidence of PNI has been on the rise in recent years (35). Despite notable advancements in the field of peripheral nerve injury repair, such as the development of microsurgical techniques, surgical repair or nerve transplantation can address nerve defects or ruptures caused by external trauma in some patients (36, 37). However, due to the limited regenerative capacity of nerve cells, surgical interventions may exacerbate damage to already impaired nerves, resulting in suboptimal functional recovery (38, 39). Consequently, there is an urgent imperative to investigate safe and effective methods for PNI treatment, presenting a formidable challenge within the domain of regenerative medicine.

The common methods of PNI repair, the diverse cell types utilized, and the mechanisms by which cell therapy facilitates PNI repair necessitate further comprehensive research. Mesenchymal stem cells (MSCs) are a type of multipotent progenitor cell that can be isolated from sources such as bone marrow, umbilical cord, and adipose tissue. These cells possess the capacity to differentiate into mesodermal and nerve cells (40, 41). MSCs are widely regarded as prime candidates for cell therapy and tissue engineering due to their unique ability to differentiate into various cell types, particularly nerve cells, both *in vitro* and *in vivo*. Currently, MSCs are being employed in clinical trials for a range of diseases (42–44). In the context of PNI repair and regeneration, MSCs have garnered extensive attention and support from researchers globally. This therapeutic approach holds broad promise because MSCs have the potential to differentiate into nerve cells and are anticipated to provide substantial support for nerve regeneration (45–47). Research in this domain has the potential to significantly improve outcomes for PNI patients, reduce disability rates, and alleviate the distress and economic burden on patients and their families (48, 49). However, more research and clinical trials are necessary to validate and refine the efficacy and safety of this treatment. Currently, research on the treatment and regeneration of peripheral nerve injuries using MSCs is in its early stages, and future studies will be pivotal in exploring the unknown related mechanisms (35, 50). Further investigation into the effects of MSCs on nerve injury repair and elucidating the mechanisms of action of MSCs will yield new insights into enhancing the repair of peripheral nerve injuries through stem cell transplantation.

Limitations of this Study include: (1) Language bias: This study only included English literature, which may lead to the exclusion of high-quality literature published in other languages. Therefore, the study results may not comprehensively cover all relevant literature in the field, which could introduce language bias. (2) Time constraints: The publication and citation frequency analysis of the literature were constrained by the time of Publication that we selected (2013–2023), which means that the papers published before 2013 and after 2024 may not be included in the analysis. This could result in relatively lower publication and citation frequency totals, affecting the comprehensiveness and accuracy of the study results. (3) Literature screening methods: Further clarification of the literature screening methods and exclusion criteria are needed to determine how to choose the criteria for literature inclusion. This can help clarify the credibility of the study and the transparency of the methods. (4) Data source selection: This study did not collect literature from multiple databases, so selection bias may exist in the data selection process. (5) Lack of in-depth analysis of publication content: Bibliometric analysis often focuses on the number and citations of publications rather than the depth and quality of literature content. This may ignore the actual contribution of the publications and the intrinsic value of the research. (6) Frequency of updates: The frequency of database updates may affect the timeliness and completeness of literature searches. If the database is not updated in real time, then the most recent research may be missed.

## 5 Conclusion

The current bibliometric analysis included 350 publications, which published by 2049 authors, distributed in 41 countries, and affiliated with 585 different institutions. This international and diverse representation reflects the complexity and attractiveness of this field. China and the Nantong University are currently the leading country and institution in this field. Although the basic research and clinical trials of MSCs based treatment of PNI have achieved remarkable results, challenges remain. Long-term follow-up and monitoring are indispensable in order to fully assess the long-term effects of MSCs in the treatment of PNI. With the continuous advancement of science and technology, we have reason to believe that these challenges will be effectively addressed, resulting in more effective MSC therapy for patients with PNI.

## Data availability statement

The datasets presented in this article are not readily available because this article followed the Preferred Reporting Items for Systematic Reviews and Meta-Analyses (PRISMA) guidelines, R-AMSTAR guidelines, as well as the Cochrane Handbook for Systematic Reviews of Interventions. Requests to access the datasets should be directed to https://clarivate.com.cn/solutions/web-of-science.

## Author contributions

AA: Data curation, Methodology, Writing – original draft, Writing – review & editing. SA: Conceptualization, Writing – original draft, Methodology. KS: Software, Writing – original draft. AM: Data curation, Formal analysis, Writing – review & editing.
